# *Salvia officinalis* L. from Italy: A Comparative Chemical and Biological Study of Its Essential Oil in the Mediterranean Context

**DOI:** 10.3390/molecules25245826

**Published:** 2020-12-10

**Authors:** Rosa Tundis, Mariarosaria Leporini, Marco Bonesi, Simone Rovito, Nicodemo G. Passalacqua

**Affiliations:** 1 Department of Pharmacy, Health and Nutritional Sciences, University of Calabria, 87036 Rende, CS, Italy; mariarosarialeporini@tiscali.it (M.L.); marco.bonesi@unical.it (M.B.); 2 History Museum of Calabria and Botanic Garden, University of Calabria, 87036 Rende, CS, Italy; simonerovito@hotmail.it (S.R.); nicodemo.passalacqua@unical.it (N.G.P.)

**Keywords:** *Salvia officinalis* L., essential oils, GC-MS, antioxidant, anticholinesterase activity, Alzheimer’s diseases

## Abstract

*Salvia officinalis* L. (sage) is one of the most appreciated plants for its plethora of biologically active compounds. The objective of our research was a comparative study, in the Mediterranean context, of chemical composition, anticholinesterases, and antioxidant properties of essential oils (EOs) from sage collected in three areas (S1–S3) of Southern Italy. EOs were extracted by hydrodistillation and analyzed by gas chromatography (GC) and gas chromatography-mass spectrometry (GC-MS). Acetylcholinesterase (AChE) and butyrylcholinesterase (BChE) inhibitory properties were investigated by employing Ellman’s method. Four in vitro assays, namely, 2,2-diphenyl-1-picrylhydrazyl (DPPH), 2,2′-azino-bis(3-ethylbenzothiazoline-6-sulfonic acid) (ABTS), ferric-reducing ability power (FRAP), and β-carotene bleaching tests, were used to study the antioxidant effects. Camphor (16.16–18.92%), 1,8-cineole (8.80–9.86%), β-pinene (3.08–9.14%), camphene (6.27–8.08%), and α-thujone (1.17–9.26%) are identified as the most abundant constituents. However, the content of these constituents varied depending on environmental factors and pedoclimatic conditions. Principal component analysis (PCA) was performed. Based on Relative Antioxidant Capacity Index (RACI), S2 essential oil exhibited the highest radical potential with an IC_50_ value of 20.64 μg/mL in ABTS test and presented the highest protection of lipid peroxidation with IC_50_ values of 38.06 and 46.32 μg/mL after 30 and 60 min of incubation, respectively. The most promising inhibitory activity against BChE was found for S3 sample (IC_50_ of 33.13 μg/mL).

## 1. Introduction

The genus *Salvia* L., one of the most important genera of the Lamiaceae family, comprises about 900 species, widespread throughout the world [1]. Some members of this genus are cultivated to be used as food spices or flavoring agents in cosmetics and perfumery.

Several species are used in traditional medicine to treat microbial infections, malaria, inflammation, and to disinfect homes after sickness [1].

*Salvia* species have attracted researchers for their biological properties, showing strong antibacterial, antifungal, anticancer, antioxidant, anticholinesterase, and anti-inflammatory effects, as well as for improvement of cognitive performance and mood [2,3,4,5].

*Salvia officinalis* L. subsp. *officinalis* (Dalmatian sage, *S. officinalis* hereafter) is a perennial, evergreen subshrub, native and endemic to the Western Balkans and the Apennine Peninsula, though it has naturalized in many places throughout the world.

*S. officinalis* (sage) is characterized by a rather high level of genetic diversity in the plastid genome as well as at the nuclear DNA level [6,7,8]. Spatial analysis of the genetic diversity of *S. officinalis* in Balkan peninsula revealed a typical pattern of isolation by distance, indicating that presumably *S. officinalis* survived in microrefugia and expanded from there resulting in secondary contact zones [9].

Southern Italian populations are at the South-West border of the distribution area of the species, representing possible differentiated populations inside the species variability.

Sage is one of the most appreciate plants for its rich essential oil (EO) and its plethora of phytochemicals extensively used in traditional medicine [10]. As its Latin gender name *Salvia* means “to cure” and species name “*officinalis*” means medicinal, it is clear that sage has a historical reputation of promoting health and treating ailments.

Several studies reported chemical analyses of its phytochemicals including essential oil, which is acknowledged worldwide because of its beneficial properties. However, it is important to take into account that the relative quantities and the presence and/or absence of some constituents are strongly affected by environmental conditions and agronomic management practices including plant genetic, elevation, topography, harvest time, as well as ecological and climatic conditions. For these reasons, essential oils (EOs) from plants collected in different countries at different seasons comprise different chemical compounds and may exert different biological effects [11]. Sage EO is used for the treatment of a range of diseases and has been shown to possess cytotoxic [12], antimutagenic [12], antimicrobial [13], antioxidant [13], and neuroprotective effects [14]. The antioxidant activity of aromatic plants has been widely explored and found to have health applications in prevention and reducing risk of diseases such as Alzheimer’s diseases (AD). This neurodegenerative disorder is the most common form of dementia, often characterized by cognitive decline and memory impairment that can affect behaviour, speech, the motor system, and orientation [15]. In AD brain, the presence of β-amyloid plaques and neurofibrillary tangles is characteristic. During AD progression, different types of neurons deteriorate, although there is a profound loss of forebrain cholinergic neurons, which is accompanied by a progressive decline in acetylcholine [16].

AD therapy is commonly based on inhibitors of acetylcholinesterase (AChE), the enzyme responsible to the hydrolysis of acetylcholine in several cholinergic pathways in the central and peripheral nervous systems. However, it was found that AChE activity remains unchanged or declines, whereas butyrylcholinesterase (BChE) activity progressively increases. Both enzymes, which differ in kinetics, substrate specificity, and activity in different brain regions, represent a useful therapeutic target for improving the cholinergic deficit responsible for the decline of cognitive and behavioral characteristics of AD. Additionally, the amyloid peptides, contained in the senile plaques, can induce inflammation in which reactive oxygen species (ROS) are produced [11]. ROS are able to damage cellular constituents and act as secondary messenger in inflammation. For these reasons, the use of compounds able of restoring the level of acetylcholine through inhibition of the AChE and BChE enzymes, eliminating ROS, and attenuating inflammatory pathways can be a multitarget strategy for the treatment and management of AD [11,17]. Several studies reported a number of new cholinesterase inhibitors isolated from medicinal plants and plant foods.

In this context, the aim of this work was to: (i) qualitatively and quantitatively analyze EOs from *S. officinalis* collected in 3 different areas of Southern Italy by gas chromatography (GC) and gas chromatography-mass spectrometry (GC-MS); (ii) compare the chemical composition of Italian sage EOs with other native Mediterranean sage EOs; (iii) investigate the potential role of sage EOs to treat neurodegenerative diseases such as AD using the in vitro cholinesterase inhibitory activity test; and (iv) evaluate the in vitro antioxidant effects.

## 2. Results and Discussion

### 2.1. Chemical Profile

The fresh aerial parts of *S. officinalis* harvested in three areas of Calabria (Southern Italy) were subjected to hydrodistillation to obtain essential oils. Two populations (S1 and S3) were on the Tyrrhenian side, whereas the third population was on the Ionia side. All sites are characterized by a Mediterranean climate, but the S2 site results with a subhumid-termo-Mediterranean bioclimate, whereas in the S1 and S3 sites, the bioclimate is humid-meso-Mediterranean. Plants grew in a shrubby habitat with an open canopy structure and some bare ground (garrigue) on a rocky limestone soil.

Essential oils were analyzed by gas chromatography (GC) and gas chromatography-mass spectrometry (GC-MS). Forty-five compounds, accounting for 96.30%, 97.56%, and 96.69% of the total composition for S1, S2, and S3, respectively, were identified in *S. officinalis* essential oils and were listed in Table 1.

**Figure 1:**
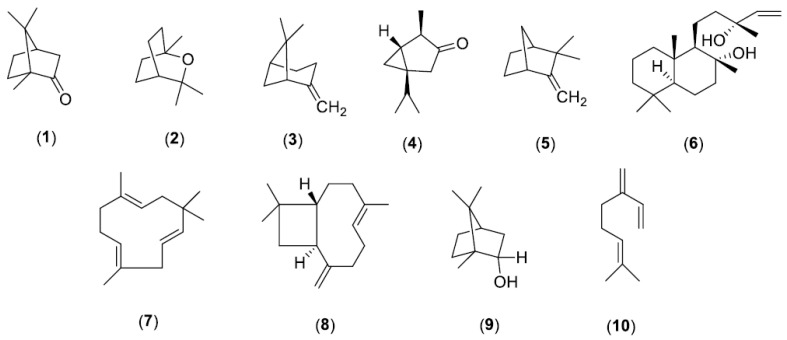
The chemical structure of the most abundant constituents of *S. officinalis* EO, camphor (**1**), 1,8-cineole (**2**), β-pinene (**3**), α-thujone (**4**), camphene (**5**), sclareol (**6**), α-humulene (**7**), trans-caryophyllene (**8**), borneol (**9**), and myrcene (**10**).

Oxygenated monoterpenes (42.06%, 40.82%, and 31.75% for S1, S3, and S2, respectively) are the dominant constituents, followed by monoterpene hydrocarbons (31.33%, 23.59%, and 20.16% for S2, S3, and S1, respectively), and sesquiterpene hydrocarbons (27.64%, 21.88%, and 20.13% for S1, S3, and S2, respectively). Camphor (16.84%) and 1,8-cineole (9.86%) are the most abundant constituents of S1 oil, followed by α-thujone (9.26%) (Figure 1). Camphor (16.16%), β-pinene (9.14%), and 1,8-cineole (8.80%) are the dominant compounds in S2 oil.

In S3 essential oil, the trend camphor (18.92%) > 1,8-cineole (9.21%) > camphene (8.08%) was observed. Other compounds such as sclareol, trans-caryophyllene, α-humulene, α-pinene, and borneol are present with percentages ranging from 2.50% to 5.15%.

Interestingly, in S2 oil, the β-pinene content was about three times higher than that found in S1 and S3. The same observation can be done for myrcene that showed a percentage higher in S2 than in S1 and S3. In S1 essential oil, the α-thujone content was about eight times higher in comparison to its content in S2. β-Cubebene and α-bergamotene are identified only in S2, while some compounds such as α-copaene, β-bourbonene, α-gurjunene, β-selinene, germacrene D spathulenol, and calarene were not identified in this essential oil. These results confirmed that the different EOs composition is subject to change under the influence of several factors including collection time, environmental factors, and climatic conditions [18].

**Figure 2:**
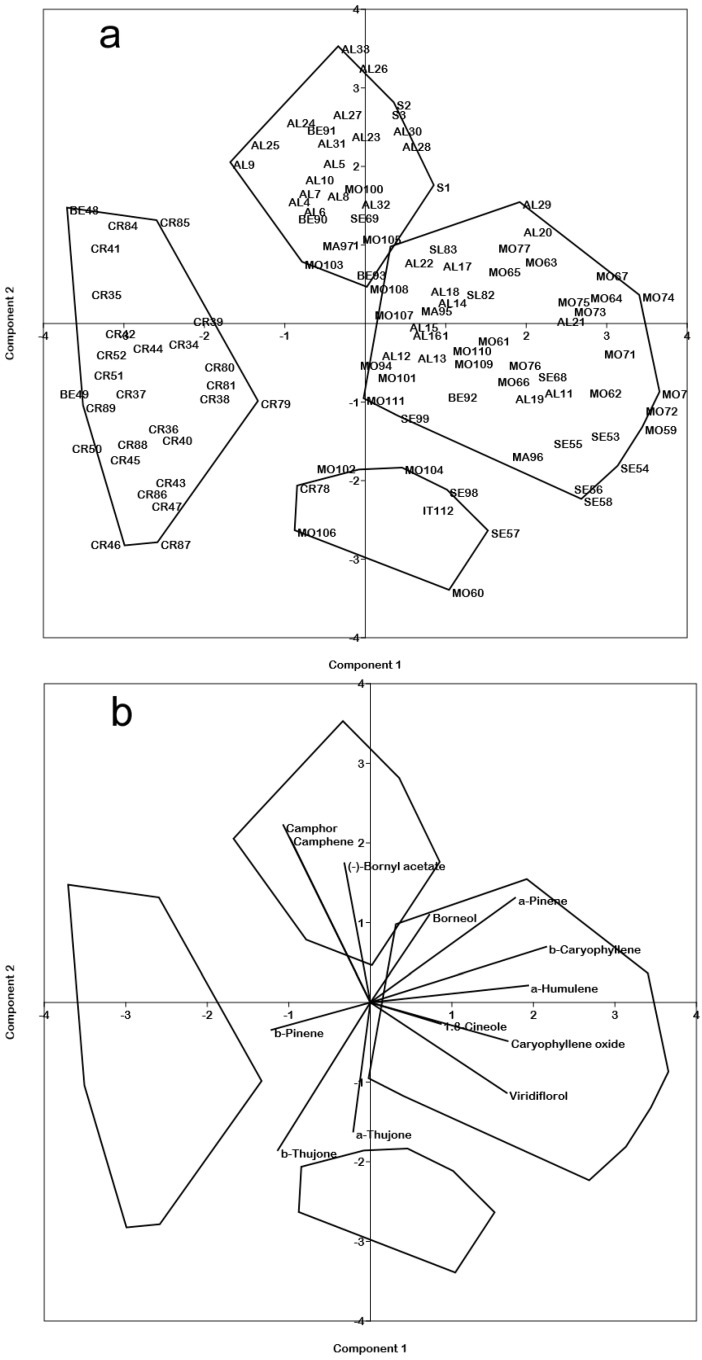
Principal component analysis (PCA) scatterplot of the first (31.23% of variability) and second (18.29%) components; (**a**) scatterplots of cases (acronyms follow the Supplementary Materials Table S1) and (**b**) scatterplot of essential oils (EOs).

In the context of the *S. officinalis* variability, principal component analysis (PCA) (Figure 2) showed on the first (31.23% of variation) and second (18.29%) principal components four more or less distinct groups. Croatian populations (CR) showed a clear separation on the left part of the scatterplot, because of a higher content in β-thujone (µ = 15.67 ± 13.28; *p* < 0.0001), and β-pinene (µ = 5.39 ± 3.087; *p* < 0.001) with respect to all other samples.

Calabrian plants (S1–S3) were on the upper part of the scatterplot, together with many samples from Albania (AL), and some from Bosnia-Erzegovina (BE), Macedonia (MA), Montenegro (MO), and Serbia (SE), sharing a higher content in camphor (µ = 27.97 ± 7.52; *p* < 0. 0001), camphene (µ = 6.29 ± 1.78; *p* < 0.0001), and bornyl acetate (µ = 2.35 ± 1.29; *p* <. 0.0001).

Noteworthy, the sample from Abruzzi (IT) was not grouped with Calabrian population, but it was in the lower part of the scatterplot, together with some samples from MO and SE, mainly because of a higher content in α-thujone (µ = 30.88 ± 12.03; *p* < 0.0001).

On the central-right part of the scatterplot, most of the samples from MO, SE, and MA, were grouped together with all samples from Slovenia (SL) and many samples from AL; they were characterized by the combination of several compounds such as 1.8-cineole (µ = 11.48 ± 3.45), viridiflorol (µ = 9.79 ± 4.58), α-humulene (µ = 7.23 ± 3.15), β-caryophyllene (µ = 6.07 ± 2.73), and α-pinene (µ = 3.35 ± 1.40).

Several minor compounds were detected only in Calabrian samples, such as α-bergamotene (S2), β-cubebene (S2), β-farnesene (S1,S3), β-selinene (S1–S3), calarene (S1,S3), germacrene D (S1,S3), manol oxide (S1–S3), and spathulenol (S1,S3). Furthermore, in S2 was detected a higher amount in myrcene, γ-muurolene, δ-cadinene, aromadendrene, and terpinolene with respect to all other samples of *S. officinalis* considered.

Previous analyses of EOs composition in indigenous populations of *S. officinalis* identified chemotypes that only partly correspond to our findings. α-Thujone and β-thujone, and their relationship, were often recognized as two compounds characterizing chemiotypes, often in contrast to camphor combined to other compounds. Jug-Dujaković et al. [9] found out three chemiotypes based on α-thujone, β-thujone, and camphor/β-pinene/borneol/bornyl acetate in Croatia.

A variation around two separate peaks of the ratio between α- and β-thujone was detected in Albania [19], separating plants of North Albania from that of South. This result is in agreement with that found by Ibraliu et al. [20], who studied seven populations from the North of Albania, showing all a very high α- to β-thujone ratio.

Studying the EO composition of *S. officinalis* in nine Balkan countries, Cvetkovikj Karanfilova et al. [21] found a high content in α-thujone, β-thujone, and camphor in a chemotype with samples from Croatia, another chemotype with Croatian samples characterized by a high content of camphor and β-pinene; a third chemotype, with the majority of the investigated populations, characterized by high content of α-thujone and camphor; and a chemotype formed by cultivated or naturalized plants with high contents in α-thujone and α-humulene.

In our analysis, β-thujone was combined to β-pinene (CR group) and to α-thujone and viridiflor (AB and few samples from MO and SE), whereas all other compounds were diversely combined in a group where it was difficult to find a clear limit between two subgroups. Calabrian populations were grouped together with a subgroup from AL for a combination in camphor, camphene, and bornyl acetate that has not been identified in other studies.

Russo et al. [12] confirmed that the variability in EOs constituents depending on environmental factors such as altitude, water availability, and pedoclimatic conditions, analyzing eighteen EOs from *S. officinalis* collected in Molise (Italy) in three different climatic macroenvironments such as lowland, low hill, and high hill. Results showed that the main components for all investigated oils are α-thujone (7.8–20.1%), camphor (8.4–20.8%), borneol (2.5–16.9%), γ-muurolene (2.9–13.8%), and sclareol (5.9–23.1%). These compounds are common constituents of the essential oil from *S. officinalis* leaves, but present with different percentages depending on sampling techniques, geographic origin, environmental factors, season, extraction methods, and genetic differences.

Similarly, Farhat et al. [22] assessed as the phenological stage influences the chemical profile of EO of *S. officinalis* collected in two different regions of the north of Tunisia, cultivated under the same conditions. 1,8-Cineole (17.6–20.4%), α-thujone (15.7–25.2%), β-thujone (5.3–7.1%), camphor (6.0–24.4%), and viridiflorol (3.1–16.3%) were the most abundant components.

The content of these volatiles varied depending on the phenological period. In particular, the highest production of 1,8-cineole was reported in the flowering period, while the major levels of camphor and viridiflorol were found at fruiting and vegetative phases of plants.

A higher content of 1,8 cineole (27.5%) and a lower content of camphor (11.5%) compared to our samples were found in the oil of *S. officinalis* from Albania [23].

Karik et al. [24] reported the EO composition of *S. officinalis* collected in Turkey. The most abundant oxygenated monoterpene was β-thujone (34.59%), followed by α-thujone (12.60%) and camphor (10.09%). A great variability was found in *S. officinalis* grown in northern India [25]. Indeed, the most abundant compounds were cis-thujone (19.8–42.5%), (*E*)-caryophyllene (1.2–16.1%), manool (3.6–15.1%), viridiflorol (3.1–12.8%), 1,8-cineole (2.8–13.8%), and camphor (1.4–22.1%).

Camphor was abundant in leaves EO, cis-thujone and manool in the stem EO, while (*E*)-caryophyllene and viridiflorol in the inflorescence oil.

Santos-Gomes and Fernandes-Ferreira [26] analyzed the essential oil from Portuguese *S. officinalis* sampled in different months of the year. From December to April, about 20% of oxygenated monoterpenes decreased, while 10% of monoterpene hydrocarbons increased. From February to April, the content of sesquiterpene hydrocarbons increased decreasing thereafter in July. Oxygenated sesquiterpenes increased in July, decreasing thereafter.

### 2.2. Anticholinesterase Activity

**Figure 3:**
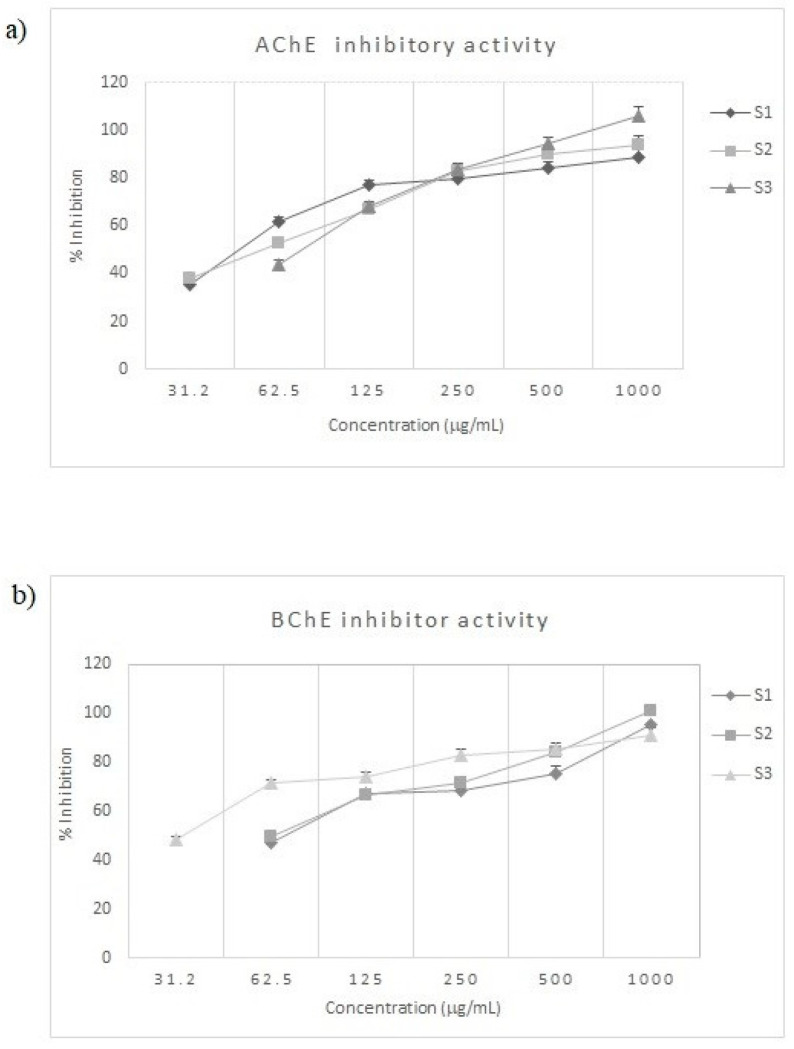
(**a**) Acetylcholinesterase (AChE) and (**b**) butyrylcholinesterase (BChE) inhibition by *S. officinalis* essential oils (S1–S3). Data are expressed as means ± S.D. (*n* = 3).

The inhibitor activity of *S. officinalis* oils against AChE and BChE was reported in Table 2 and Figure 3. These enzymes play a significant role in decreasing choline levels in the body representing a therapeutic strategy to treat AD [27].

EOs have become of great interest, due to their availability, few side effects and toxicity, as well as their biodegradability [28]. Moreover, EOs constituents are able to cross the blood–brain barrier due to their small molecular size and lipophilicity. Sage EOs showed a good AChE and BChE inhibitory activity (Table 2). The highest activity against AChE was exhibited by S1 with an IC_50_ values of 47.68 μg/mL followed by S2 (IC_50_ of 47.68 μg/mL). Comparing the results, several differences were displayed. Indeed, the S1 inhibitory activity was 1.6-times higher in comparison to S3. This action can be justified for highest content in β-pinene and minor constituent borneol. Pearson’s correlation (Supplementary Materials, Table S2) demonstrated that these compounds were positively correlated with AChE (r = 0.99 and 0.96 for β-pinene and borneol, respectively).

Other compounds, namely, α-phellandrene, linalool, trans-caryophyllene, exhibited a significant positive correlation with AChE inhibition (r = 0.99). It is possible that the activity of the most abundant compounds is modulated by constituents present in the EOs in smaller quantities. This is probably due to the ability of these compounds (i) to penetrate cells, (ii) to make a lipophilic or hydrophilic linkage, and (iii) to fix on cell wall [29].

The strongest inhibitory ability against BChE was observed for S3 with an IC_50_ values of 33.13 μg/mL. This activity is 2.1-times higher in comparison to S1. Pearson’s correlation showed that camphor, that showed a higher content in S3 compared to S1, positively correlated with BChE (r = 0.82). The main difference between AChE and BChE is the presence of subregions within the gorge, which includes an acyl-binding pocket and a peripheral anionic site. The differences observed in the acyl-binding site are especially important. Indeed, in AChE the acyl-binding site presented two aromatic amino acids Phe295 and Phe297, which are, respectively, displaced by aliphatic amino acids Leu286 and Val288 in BChE [30,31]. These structural differences between the active sites of these enzymes may justify the different inhibitory activities of S1 and S3.

Our data are in according with literature data. *S. officinalis* EO from Tunisia exhibited notable activity against AChE with IC_50_ value of 38.71 mg/L [32].

A less activity was reported for *S. officinalis* EO from Colombia that showed an anti-AChE activity with an IC_50_ value of 78 mg/L [33] and much lower IC_50_ values ranging between 326.7 and 867.4 mg/L were found by Cutillas et al. [13] for four Spanish *S. officinalis* EOs. Previously, Albano et al. [34] reported more interesting results against AChE (IC_50_ of 50.8 mg/L) than that reported by Orhan et al. [35] (63.8% at a concentration 1 mg/mL) and by Ferreira et al. [11] (46% of inhibition at a concentration of 0.5 mg/mL) for *S. officinalis* essential oils collected in Portugal.

EOs from other Salvia species have been intensively investigated as potential source of neuroprotective agents. Kennedy et al. [36] and Temel et al. [37], indicated that *S. lavandulaefolia* and *S. pseudeuphratica* EOs were highly potent inhibitor of AChE, with an IC_50_ value of 3 and 26.00 μg/mL, respectively. The inhibitory potency of *S. lavandulaefolia*, collected in different bioclimatic zone in the south-east of Spain, was also investigated by Cutillas et al. [38]. IC_50_ ranging from 108.0 to 142.4 mg/L were obtained against AChE. An in vivo study carried out by Perry et al. [39] confirmed this inhibition.

*S. lavandulaefolia* EO given orally once daily for 5 days to rats decreased striatal AChE activity at a lower tested dose (20 μL) and striatal and hippocampal AChE activity at a higher tested dose (50 μL); at both doses, there was no change in the AChE activity in the cortex. Temel et al. [37] and Bahadori et al. [40] demonstrated also the neuroprotective ability of *S. hydrangea*, *S. divaricata*, and *S. nemorosa*. In comparison to *S. divaricata* and *S. nemorosa*, the specie *S. hydrangea* was more efficient against AChE (IC_50_ of 64.68, 434.1, and 40.0, mg/L, respectively).

*S. fruticosa* essential oil showed lower inhibition against AChE with percentage of 37.0% and 39.2% at a concentration of 1 mg/mL for two Italian samples [41], 26.04% at concentration of 50 mg/L for a Turkish sample [42], and 73.52% at 100 μg/mL for another Turkish sample [43]. Interesting cholinesterases inhibitory activity was found for *S. leriifolia* EO from in Iran (IC_50_ of 0.32 and 0.29 μL/mL for AChE and BChE, respectively) [44]. *S. officinalis* and *S. sclarea* EOs displayed a notable inhibition towards BChE having 66.3% and 76.0% inhibition, respectively [34]. An inhibition percentage of 51.24% and 22.73% at 100 μg/mL was found against BChE for Turkish *S. fruticosa* [42,43], while two Italian EOs were not able to inhibit this enzyme [34]. The EOs activity likely results from a complex interaction of its compounds, ultimately producing synergistic or antagonistic responses [45,46]. Among EOs constituents, α-pinene and 1,8-cineole inhibited AChE with IC_50_ values of 0.63 and 0.67 mM, respectively [47]. In other studies, camphor and β-pinene presented an IC_50_ value of 21.43 µM and an inhibition percentage of 48.5% (at 1.0 mM), respectively [48,49]. Interestingly, selective activity of trans-caryophyllene was observed by Bonesi et al. [45] against BChE (IC_50_ of 78.6 μM). Some clinical trials suggested that sage EOs can lead to improvements of the mood and cognition [2]. Moss et al. [50] evaluated the potential of the aromas of *S. officinalis* and *S. lavandulaefolia* EO to affect cognition and mood in 135 healthy volunteers. Only *S. officinalis* aroma produced a significant enhancement of memory quality. Kennedy et al. [36] reported improvement of cognitive performance and mood after *S. lavandulaefolia* EO oral consumption (capsules containing 50 μL of EO) in 36 healthy participants. Substantial improvements in some aspects of mood were also recorded by Tildesley et al. [51] after *S. lavandulaefolia* EO treatment (50 μL) in 24 healthy participants. In another study, the administration of 50 μL of *S. lavandulaefolia* EO to patients affected by AD induced a reduction of neuropsychiatric symptoms and an improvement in attention [16].

### 2.3. Antioxidant Activity

Different methods are available to examine the antioxidant capacity of a matrix. Taking into account the high complexity of composition of an EO, herein the antioxidant properties of sage EOs were investigated by applying 4 in vitro tests, such as ABTS, DPPH, FRAP, and β-carotene bleaching tests. Data are reported in Table 3.

An antioxidant activity in a concentration-dependent manner was found for all EOs. Except for FRAP test, S3 oil showed the best antioxidant activity. Indeed, S2 sample exhibited the highest radical potential with IC_50_ values of 20.64 μg/mL in ABTS test and a percentage of 35.33% in DPPH test. Additionally, this EO presented the highest protection of lipid peroxidation with IC_50_ values of 38.06 and 46.32 μg/mL after 30 and 60 min of incubation, respectively. In iron reduction capacity, S1 was the most active. Significant correlations (Table S2) were found between α-pinene content and DPPH test (r = 1.00). A positive correlation between myrcene and β-pinene content and ABTS test (r = 1.00 and 0.99, respectively) was also found. In addition, the Pearson’s correlation coefficient was positive between calarene, manoyl oxide, and manool and β-carotene blanching test after 30 min of incubation with r = 1.00, and 0.99, respectively. Moreover, caryophyllene oxide and terpinene-4-ol were positively correlated with β-carotene blanching test after 60 min of incubation (r = 0.99 and 0.98, respectively). In FRAP test, the higher positive correlation was found with γ-terpinene (r = 0.99).

**Figure 4:**
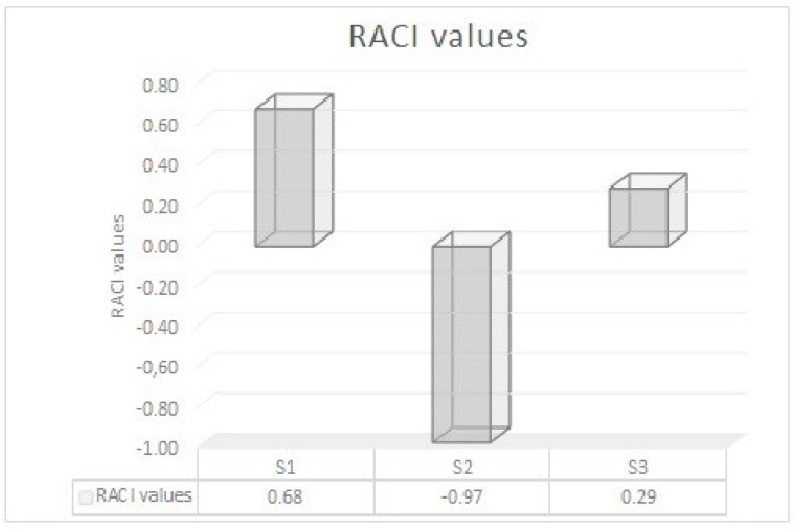
Relative Antioxidant Capacity Index (RACI) values of *S. officinalis* essential oils (S1–S3).

The Relative Antioxidant Capacity Index (RACI) value of each *S. officinalis* EO was calculated. Based on RACI data, the following antioxidant rank order has been found: S2 > S3 > S1 (Figure 4).

According to Michelina et al. [52], the Italian sage demonstrated a more promising radicals scavenging activity with an ID_50_ value of 299.1 and 101.5 μg/mL for DPPH and ABTS tests, respectively. For *S. officinalis* leaves EO from Tunisia, an IC_50_ value of 8.31 mg/L was reported against DPPH radicals [32]. This result was comparable with another Turkish sage EO previously analyzed by Bouaziz et al. [53] that found an IC_50_ value of 7.70 mg/L. The capacity to scavenge DPPH radicals was also demonstrated for sage leaves collected in Morocco [54] with an IC_50_ of 309.42 mg/mL.

Interestingly, Boutebouhart et al. [55] compared the antioxidant activity of *S. officinalis* leaves EO cultivated in Algeria and obtained by microwave-assisted hydrodistillation, conventional hydrodistillation technique, and steam distillation. The EOs obtained by steam distillation and hydrodistillation presented the highest percentage of DPPH radical inhibition with values of 40.25% and 36.75%, respectively, (at 1000 mg/L).

Four EOs from *S. officinalis* from Spain were analyzed by Cutillas et al. [13] for their antioxidant activity against free radicals as well as ferric reducing and ferrous chelating agents. In the oxygen radical absorbance capacity (ORAC) and ABTS test, EO showed a range of activity of 98.8–154.9 and 0.6–1.2 mg Trolox equivalent/g EO, respectively. In DPPH test, all EOs presented a value of 0.1 mg TE/g EO. In thiobarbituric acid reactive substances (TBARS) test, a range of 0.5–1.2 mg butylhydroxytoluene equivalent/g EO was reported. A similar value was observed for their capacity to reduce ferric ions and chelating activity.

Several EO constituents were recently investigated by Nie et al. [56] for their potential antioxidant activity. In ABTS test, the following order of activity was found: o-cymene > camphene > α-pinene > camphor > bornyl acetate > α-bisabolene. In DPPH test, the order of activity was: camphor > bornyl acetate > p-cymene > 3-carene > o-cymene > α-pinene > terpinen-4-ol > camphene > linalool oxide acetate > β-pinene > α-bisabolene. Authors speculated that in DPPH test, a carbonyl group and a double bond conjugated to the carbonyl group seem to play an important role in the antioxidant activity. Instead, in ABTS test, the cyclic ether group is important for the founded activity.

## 3. Materials and Methods

### 3.1. Chemicals and Reagents

Solvents of analytical grade were purchased from VWR International s.r.l. (Milan, Italy). Propyl gallate, ascorbic acid, 2,2-azinobis(3-ethylbenzothiazoline-6-sulfonic) acid (ABTS) solution, butylated hydroxytoluene (BHT), 2,2-diphenyl-1-picrylhydrazyl (DPPH), tripyridyltriazine (TPTZ), β-carotene, linoleic acid, Tween 20, physostigmine, acetylcholinesterase (AChE) from *Electrophorus electricus* (EC 3.1.1.7, Type VI-S) 5,5′-dithio-bis(2-nitrobenzoic acid) (DTNB), butyrylcholinesterase (BChE) from equine serum (EC 3.1.1.8), acetylthiocholine iodide (ATCI), and butyrylthiocholine iodide (BTCI) were obtained from Sigma-Aldrich S.p.a. (Milan, Italy).

### 3.2. Plant Materials

*Salvia officinalis* aerial parts were harvested in June 2018 in Calabria (Southern, Italy) in Orsomarso (sample S1, 39°48′5.16″ N, 15°54′43.63″ E, 2400 m a.s.l.; voucher specimen n. CLU 26259), Civita (sample S2, 39°49′41.74″ N, 16°18′14.36″ E, 620 m a.s.l.; voucher specimen n. CLU 26262), and Buonvicino (sample S3, 39°41′24.49″ N, 15°55′49.90″ E, 6500 m a.s.l.; voucher specimen n. CLU 26265). The authentication was carried out by Dr. N.G. Passalacqua at the Natural History Museum of Calabria and the Botanic Garden, University of Calabria.

### 3.3. Isolation of Essential Oils

The fresh aerial parts of *S. officinalis* were subjected to hydrodistillation for 3 h using a Clevenger-type apparatus [57] to obtain essential oils with yields of 0.35%, 0.37%, and 0.45%, for S1, S2, and S3, respectively. The white-yellow EOs were dried over anhydrous sodium sulfate, and stored under N_2_ at +4 °C in brown glass bottles until tested and analyzed.

### 3.4. Gas Chromatography (GC) and Gas Chromatography-Mass Spectrometry (GC-MS) Analyses

The chemical composition of *S. officinalis* EOs was investigated using a Hewlett-Packard gas chromatograph (Agilent, Milan, Italy) equipped with an HP-5 capillary column (30 m × 0.25 mm, 0.25 μm), associated with a Hewlett-Packard mass spectrometer (Agilent, Milan, Italy) (GC-MS) using electron impact ionization (EI) carried out at 70 eV as previously reported [58]. Helium was used as carrier gas. Samples were also analyzed using a Shimadzu GC17A gas chromatograph (GC) (Shimadzu, Milan, Italy) equipped with an ionization flame detector (FID) and an HP-5 capillary column (30 m × 0.25 mm, 0.25 μm). Nitrogen was used as carrier gas. Sage EOs constituents were tentatively identified by comparing their Retention Indices (RI) either with those in the literature or with those of standards [59,60]. RI were calculated under the same operating conditions in relation to a homologous series of *n*-alkanes (C_8_–C_24_).

### 3.5. In Vitro Cholinesterases Inhibitory Activity

AChE and BChE inhibitory properties of *S. officinalis* EOs were evaluated by applying the Ellman’s method as previously reported [45]. AChE from *Electrophorus electricus* (EC 3.1.1.7, Type VI-S) and BChE from equine serum (EC 3.1.1.8) were used. Acetylthiocholine iodide and butyrylthiocholine iodide were employed as the reaction substrates. Physostigmine was the positive control. The absorbance was read at 405 nm.

### 3.6. Antioxidant Properties

Four in vitro tests, namely, 2,2-diphenyl-1-picrylhydrazyl (DPPH), 2,2′-azino-bis(3-ethylbenzothiazoline-6-sulfonic acid) (ABTS), ferric-reducing ability power (FRAP), and β-carotene bleaching assays, were used to assess the antioxidant effects of *S. officinalis* essential oils as previously described [61]. DPPH and ABTS tests were applied to examine the radicals scavenging effects of sage essential oils. In DPPH assay, samples were tested at different concentrations in the range of 62.5–1000 μg/mL. Absorbance was measured at 517 nm and ascorbic acid was used as positive control. In ABTS assay, samples were analyzed at concentrations ranging from 1 to 400 μg/mL. The absorbance was read at 734 nm. Ascorbic acid was used as positive control.

FRAP test was used to evaluate the ability of sage EOs to reduce iron ions. FRAP reagent was prepared by mixing tripyridyltriazine, FeCl_3_, acetate buffer, and HCl. Absorbance was read at 595 nm. In addition, butylated hydroxytoluene (BHT) was used as a positive control.

β-Carotene bleaching test was applied to investigate the potential ability of sage EOs to inhibit lipids peroxidation using propyl gallate as a positive control. The antioxidant activity (AA) was calculated using the equation: AA = [(A − A_t_)/(A* − A_t_*)] × 100 where A and A* are the absorbance values at the time 0 for samples and control, respectively, and A_t_ and A_t_* are the absorbance values after 30 and 60 min of incubation for samples and control, respectively.

### 3.7. Statistical Analysis

Experiments were carried out in triplicate. Data are reported as means ± standard deviation (S.D.). The concentration giving 50% inhibition (IC_50_) was calculated by nonlinear regression with the use of Prism GraphPad Prism version 4.0 for Windows (GraphPad Software, San Diego, CA, USA). Data were statistically analyzed using one-way ANOVA followed by Dunnett’s post hoc test for multiple comparisons with control and Tukey’s test to determine any significant difference on chemical parameters. Differences were considered to be significant at *p* < 0.05 in the biological tests. Relative Antioxidant Capacity Index (RACI) is an integrated statistical application to evaluate the antioxidant capacity values generated by different tests [62]. RACI values were calculated using the following equation: RACI = (x − μ)/σ, where x is the raw data, μ is the mean, and σ is the standard deviation.

Studies of the Pearson’s correlation coefficient (r) and linear regression, assessment of repeatability, calculation of average, and relative standard deviation were performed using Microsoft Excel 2010 software. Significant levels were defined at *p* < 0.05, *p* < 0.01, and *p* < 0.001. All these analyses were performed by GraphPad Prism.

Literature data on EOs composition of natural populations of *S. officinalis* (Supplementary Materials, Table S1) were collected and organized in a 120 variables (compounds) per 112 cases (plant samples) matrix. Average value (µ) and standard deviation were reported, when opportune. The most representative EO (detected in over 90% of cases) were selected and tested under normality (Shapiro–Wilk). EOs that did not fit the test (*p* < 0.05) were log transformed (Ln (x + 1)) and tested again under normality and, when the test was not fitted, distance from normality was checked; EOs with skewness over 1 were excluded. Variables were standardized (z-score) and principal component analysis (PCA) was performed. The first and the second component were considered and cases were grouped based on the minimal spanning tree (Euclidean distance). EOs were subject to univariate analysis (*t*-test and F-test) to check differences in essential oil content among groups; probability test (P) was reported. Statistical analyses were performed using PAST 4.1 software (Copyright Hammer & Harper; free download from: https://www.nhm.uio.no/english/research/infrastructure/past/).

## 4. Conclusions

In conclusion, in this work, three sage EOs (S1–S3) from Southern Italy (Calabria) were chemically and biologically investigated. The interest in these species is due to the fact that Southern Italian populations are at the South-West border of the distribution area of the species, representing possible differentiated populations inside the species variability.

Both qualitative and quantitative analyses of *S. officinalis* essential oils revealed that oxygenated monoterpenes are the dominant classes of constituents in which camphor and 1,8 cineole are the most abundant. However, some minor constituents, such as α-bergamotene (S2), β-cubebene (S2), β-farnesene (S1,S3), β-selinene (S1–S3), calarene (S1,S3), germacrene D (S1,S3), manol oxide (S1–S3), and spathulenol (S1,S3), were detected only in Calabrian samples. Moreover, a higher amount of myrcene, γ-muurolene, δ-cadinene, aromadendrene, and terpinolene was found in S2 essential oil with respect to all other samples of *S. officinalis*.

Herein, we confirmed the in vitro antioxidant and neuroprotective effects of *S. officinalis* essential oils. The most promising health properties were observed for S2 essential oil in antioxidant tests, while S1 and S3 samples exhibited a significant potential inhibitory activity against AChE and BChE enzymes. Further in vivo studies are needed in order to establish synergism and antagonism effects, route of administration, and dose in order to prospect a potential use of these EOs as new drugs.

EOs from sage collected in different areas of Calabria represent as potential source of bioactive molecules with anticholinesterase and antioxidant properties useful for the treatment and management of neurodegenerative disorders such as AD.

## Figures and Tables

**Table 1:** The main identified constituents (%) of *S. officinalis* essential oils (EOs).

Compound	RI ^a^		%		I.M ^b^	Sign.
		S1	S2	S3		
Thujene	926	0.46 ^a^ ± 0.05	0.22 ^b^ ± 0.03	tr	1,2	**
α-Pinene	938	3.78 ± 0.43 ^c^	4.66 ± 0.17 ^a^	4.34 ± 0.36 ^b^	1,2,3	**
Camphene	953	6.27 ± 0.74 ^c^	7.53 ± 0.34 ^b^	8.08 ± 0.55 ^a^	1,2,3	**
Sabinene	973	0.45 ± 0.05 ^a^	0.18 ± 0.07 ^b^	nd	1,2,3	**
β-Pinene	980	3.08 ± 0.42 ^c^	9.14 ± 1.66 ^a^	3.64 ± 0.21 ^b^	1,2,3	**
Myrcene	993	1.31 ± 0.14 ^c^	4.86 ± 0.57 ^a^	2.02 ± 0.20 ^b^	1,2,3	**
α-Phellandrene	1005	0.09 ± 0.02 ^c^	0.14 ± 0.04 ^b^	0.27 ± 0.05 ^a^	1,2	**
α-Terpinene	1012	0.24 ± 0.03	0.20 ± 0.01	0.22 ± 0.06	1,2,3	ns
*p*-Cymene	1025	0.17 ± 0.02 ^b^	0.14 ± 0.03 ^b^	0.23 ± 0.03 ^a^	1,2,3	**
Limonene	1030	1.78 ± 0.14 ^c^	1.92 ± 0.56 ^b^	2.42 ± 0.01 ^a^	1,2,3	**
1,8-Cineole	1034	9.86 ± 1.43 ^a^	8.80 ± 1.04^c^	9.21 ± 1.32 ^b^	1,2,3	**
(*Z*)-β-Ocimene	1038	0.48 ± 0.01 ^b^	0.63 ± 0.01 ^a^	0.13 ± 0.03 ^c^	1,2	**
(*E*)-β-Ocimene	1049	0.18 ± 0.04 ^b^	0.22 ± 0.01 ^a^	0.10 ± 0.01 ^b^	1,2	**
γ-Terpinene	1057	0.68 ± 0.11 ^a^	0.35 ± 0.01 ^c^	0.50 ± 0.02 ^b^	1,2,3	**
Terpinolene	1086	1.19 ± 0.18 ^b^	1.14 ± 0.07 ^b^	1.64 ± 0.13 ^a^	1,2,3	**
Linalool	1098	tr	0.27 ± 0.02 ^b^	0.99 ± 0.07 ^a^	1,2,3	**
α-Thujone	1106	9.26 ± 1.10 ^a^	1.17 ± 0.04 ^c^	7.63 ± 0.01 ^b^	1,2	**
Camphor	1145	16.84 ± 2.67 ^b^	16.16 ± 2.54 ^c^	18.92 ± 2.76 ^a^	1,2	**
Borneol	1167	4.48 ± 0.18 ^b^	4.68 ± 0.54 ^a^	2.34 ± 0.11 ^c^	1,2	**
Terpinen-4-ol	1176	0.56 ± 0.06 ^b^	0.44 ± 0.05 ^c^	0.74 ± 0.01 ^a^	1,2	**
α-Terpineol	1189	1.06 ^a^ ± 0.08	0.23 ± 0.04 ^b^	0.99 ± 0.07 ^a^	1,2,3	**
(–)-Bornyl acetate	1286	1.56 ± 0.14 ^b^	1.17 ± 0.10 ^c^	4.09 ± 0.01 ^a^	1,2	**
α-Cubebene	1352	0.51 ± 0.06 ^c^	0.64 ± 0.06 ^a^	0.66 ± 0.05 ^a^	1,2	**
α-Ylangene	1373	0.51 ± 0.04 ^b^	1.90 ± 0.22 ^a^	0.35 ± 0.01 ^c^	1,2	**
α-Copaene	1377	0.81 ± 0.06 ^a^	nd	0.73 ± 0.06 ^a^	1,2	**
β-Cubebene	1382	nd	1.09 ± 0.08 ^a^	nd	1,2	**
β-Bourbonene	1385	1.20 ± 0.13 ^a^	nd	1.23 ± 0.43 ^a^	1,2	**
α-Bergamotene	1403	nd	1.30 ± 0.06 ^a^	nd	1,2	**
α-Gurjunene	1407	0.12 ± 0.01 ^b^	nd	0.34 ± 0.01 ^a^	1,2	**
*trans*-Caryophyllene	1415	4.53 ± 0.15 ^c^	4.73 ± 0.23 ^b^	4.96 ± 0.14 ^a^	1,2,3	**
Aromadendrene	1437	1.00 ± 0.06 ^b^	2.31 ± 0.10 ^a^	0.84 ± 0.06 ^c^	1,2	**
β-Farnesene	1441	nd	0.86 ± 0.04 ^a^	0.67 ± 0.01 ^b^	1,2	**
α-Humulene	1455	3.91 ± 0.32 ^a^	3.41 ± 0.64 ^b^	3.10 ± 0.01 ^c^	1,2	**
*allo*-Aromadendrene	1463	0.34 ± 0.03 ^c^	1.15 ± 0.08 ^a^	0.46 ± 0.06 ^b^	1,2	**
β-Selinene	1475	0.20 ± 0.02 ^a^	nd	0.24 ± 0.01 ^a^	1,2	**
Germacrene D	1477	0.21 ± 0.04 ^a^	nd	0.19 ± 0.02 ^a^	1,2	**
γ-Muurolene	1478	1.08 ± 0.15 ^b^	3.76 ± 0.32 ^a^	0.25 ± 0.01 ^c^	1,2	**
γ-Cadinene	1515	0.85 ± 0.07 ^b^	0.92 ± 0.05 ^a^	0.87 ± 0.01 ^b^	1,2	**
δ-Cadinene	1526	1.30 ± 0.01 ^b^	2.40 ± 0.23 ^a^	0.97 ± 0.22 ^c^	1,2	**
Spathulenol	1578	0.64 ± 0.03 ^a^	nd	0.30 ± 0.01 ^b^	1,2	**
Caryophyllene oxide	1580	0.23 ± 0.01 ^a^	0.17 ± 0.05 ^b^	0.28 ± 0.04 ^a^	1,2	**
Viridiflorol	1591	4.13 ± 0.54 ^a^	3.30 ± 0.24 ^b^	2.90 ± 0.01 ^c^	1,2	**
Calarene	1629	2.42 ± 0.11 ^a^	nd	1.77 ± 0.11 ^b^	1,2	**
Manoyl oxide	1989	0.97 ± 0.06 ^a^	0.23 ± 0.01 ^c^	0.88 ± 0.01 ^b^	1,2	**
Manool	2055	2.41 ± 0.43 ^a^	1.12 ± 0.13 ^c^	2.23 ± 0.13 ^b^	1,2	**
Sclareol	2226	5.15 ± 0.54 ^a^	4.16 ± 0.24 ^b^	3.97 ± 0.01 ^c^	1,2	**
Total		96.30	97.56	96.69		

S1: *S. officinalis* from Orsomarso; S2: *S. officinalis* from Civita; S3: *S. officinalis* from Buonvicino. Data are expressed as the mean ± standard deviation (*n* = 3). ^a^ RI: Retention indices on HP-5 MS column. ^b^ I.M, identification method: (1): comparison of retention times; (2): comparison of mass spectra with MS libraries, (3): comparison with authentic compounds; tr: trace (<0.1%). nd: not detected. Differences were evaluated by one-way analysis of variance (ANOVA) completed with a multicomparison Tukey’s test; ** *p* < 0.05. Means in the same row with different small letters differ significantly (*p* < 0.05). Sign: significant; ns: not significant.

**Table 2:** In vitro anticholinesterase activity (IC_50_, µg/mL) of *S. officinalis* essential oils.

	AChE	BChE	SI (BChE/AChE)
**Sample**			
S1	47.68 ± 1.81 ****	70.94 ± 2.80 ****	1.48
S2	58.35 ± 2.05 ****	63.43 ± 2.43 ****	1.08
S3	77.51 ± 2.91 ****	33.13 ± 1.33 ****	0.42
Physostigmine	0.11 ± 0.01	0.21 ± 0.03	2.0

S1: *S. officinalis* from Orsomarso; S2: *S. officinalis* from Civita; S3: *S. officinalis* from Buonvicino. Data are expressed as means ± S.D. (*n* = 3). SI: Selective Index. Differences within and between groups were evaluated by one-way ANOVA followed by a multicomparison Dunnett’s test α = 0.05): **** *p* < 0.0001 compared with the positive control (physostigmine).

**Table 3:** In vitro antioxidant activity of sage essential oils.

Sample	DPPH Test IC_50_ (µg/mL)	ABTS Test IC_50_ (µg/mL)	β-Carotene Bleaching Test IC_50_ (µg/mL)		FRAP Test μM Fe (II)/g
			t 30 min	t 60 min	
*S. officinalis*					
S1	31.58% ^a^	39.63 ± 3.43 ****	54.81 ± 3.43 ****	59.69 ± 3.66 ****	3.11 ± 1.61 ****
S2	35.33% ^a^	20.64 ± 1.90 ****	38.06 ± 2.28 ****	46.32 ± 2.74 ****	0.73 ± 0.09 ****
S3	32.52% ^a^	24.52 ± 2.67 ****	50.07 ± 3.09 ****	70.25 ± 3.93 ****	1.56 ± 1.02 ****
Positive Control					
Ascorbic acid	5.02 ± 0.80	1.71 ± 0.06			
Propyl gallate			0.09 ± 0.004	0.09 ± 0.004	
BHT					63.22 ± 4.3

S1: *S. officinalis* from Orsomarso; S2: *S. officinalis* from Civita; S3: *S. officinalis* from Buonvicino. Data are expressed as means ± S.D. (*n* = 3). ^a^ At concentration of 1000 μg/mL. Differences within and between groups were evaluated by one-way ANOVA followed by a multicomparison Dunnett’s test (α = 0.05): **** *p* < 0.0001 compared with the positive controls.

## Supplementary Materials

The following are available online, Table S1: Acronyms, country, and sample number used for PCA. Table S2: Pearson’s correlation between EO constituents and biological activities.
