# Supplementary material for: Salvia officinalis L. from Italy: A Comparative Chemical and Biological Study of Its Essential Oil in the Mediterranean Context
Source: Molecules. 2020 Dec 10;25(24):5826. doi: 10.3390/molecules25245826 (PMC7763040; doi:10.3390/molecules25245826)
Supplement: Supplementary file 1 [file molecules-25-05826-s001.pdf]

Supplementary Materials to accompany:

Supplementary material S1

Acronym	Country	Sample Number	Publication
AL4	Albania	P1	
AL5	Albania	P2	[1]
AL6	Albania	P3	
AL7	Albania	P4	
AL8	Albania	P5	
AL9	Albania	P6	
AL10	Albania	P7	
AL11	Albania	1	
AL12	Albania	2	
AL13	Albania	3	
AL14	Albania	4	
AL15	Albania	5	
AL16	Albania	6	
AL17	Albania	7	
AL18	Albania	8	
AL19	Albania	9	
AL20	Albania	10	
AL21	Albania	11	[2]
AL22	Albania	12	
AL23	Albania	13	
AL24	Albania	14	
AL25	Albania	15	
AL26	Albania	16	
AL27	Albania	17	
AL28	Albania	18	
AL29	Albania	19	
AL30	Albania	20	
AL31	Albania	21	
AL32	Albania	22	
AL33	Albania	23	
CR34	Croazia	P01	
CR35	Croazia	P02	
CR36	Croazia	P03	

CR37	Croazia	P05	[3]
CR38	Croazia	P07	
CR39	Croazia	P08	
CR40	Croazia	P09	
CR41	Croazia	P10	
CR42	Croazia	P11	
CR43	Croazia	P12	
CR44	Croazia	P15	
CR45	Croazia	P16	
CR46	Croazia	P17	
CR47	Croazia	P18	
BE48	Bosnia ed Herzegovina	P19	
BE49	Bosnia ed Herzegovina	P20	
CR50	Croazia	P21	
CR51	Croazia	P22	
CR52	Croazia	P25	
SE53	Serbia	P01_Fl_WD	[4]
SE54	Serbia	P01_Fl_SD	
SE55	Serbia	P01_FG_WD	
SE56	Serbia	P01_FG_SD	
SE57	Serbia	P01_ST_WD	
SE58	Serbia	P01_ST_SD	
MO59	Montenegro	A-1_Fg	[5]
MO60	Montenegro	B-1_Fg	
MO61	Montenegro	B-2_Fg	
MO62	Montenegro	B-3_Fg	
MO63	Montenegro	B-4_Fg	
MO64	Montenegro	B-5_Fg	
MO65	Montenegro	B-6_Fg	
MO66	Montenegro	C-1_Fg	
MO67	Montenegro	C-2_Fg	
SE68	Serbia	D-1_Fg	
SE69	Serbia	D-2_Fg	
MO70	Montenegro	A-1_Fl	
MO71	Montenegro	B-2_Fl	
MO72	Montenegro	B-3_Fl	
MO73	Montenegro	B-4_Fl	
MO74	Montenegro	B-5_Fl	
MO75	Montenegro	B-6_Fl	
MO76	Montenegro	C-1_Fl	
MO77	Montenegro	C-2_Fl	

CR78	Croazia	79	[6]
CR79	Croazia	101	
CR80	Croazia	109	
CR81	Croazia	126	
SL82	Slovenia	P01	
SL83	Slovenia	P02	
CR84	Croazia	P03	
CR85	Croazia	P04	
CR86	Croazia	P05	
CR87	Croazia	P06	
CR88	Croazia	P07	
CR89	Croazia	P08	[7]
BE90	Bosnia ed Herzegovina	P09	
BE91	Bosnia ed Herzegovina	P10	
BE92	Bosnia ed Herzegovina	P11	
BE93	Bosnia ed Herzegovina	P12	
MO94	Montenegro	P13	
MA95	Macedonia	P14	
MA96	Macedonia	P15	
MA97	Macedonia	P16	
SE98	Serbia	P17	
SE99	Serbia	P18	
MO100	Montenegro	P01	
MO101	Montenegro	P02	
MO102	Montenegro	P03	
MO103	Montenegro	P04	[8]
MO104	Montenegro	P05	
MO105	Montenegro	P06	
MO106	Montenegro	P07	
MO107	Montenegro	P08	
MO108	Montenegro	P09	
MO109	Montenegro	P10	
MO110	Montenegro	P11	
MO111	Montenegro	P12	
IT112	Italia		[9]

Supplementary material S2.

Pearson's correlation between essential oil constituents and biological activities.

	AChE Test	BChE Test	DPPH Test	ABTS Test	β-carotene Test		FRAP Test
					30 min	60 min	
Thujene	−0.98 ***	0.94 ***	−0.26^{ns}	−0.22 ^{ns}	0.30 ^{ns}	−0.42 ^{ns}	0.66 ^{ns}
α-Pinene	0.50 ^{ns}	−0.34 ^{ns}	1.00***	0.88 **	−0.92 ***	−0.42 ^{ns}	−1.00 ***
Camphene	0.93 ***	−0.85 **	0.45^{ns}	0.41 ^{ns}	−0.48 ^{ns}	0.23 ^{ns}	−0.80 *
Sabinene	−0.96 ***	0.90 ***	−0.35^{ns}	−0.30 ^{ns}	0.38 ^{ns}	−0.33 ^{ns}	0.73 ^{ns}
β-Pinene	0.99 ^{ns}	0.25 ^{ns}	0.99***	0.99 ***	−0.98 ***	−0.86 **	−0.82 **
Myrcene	0.04 ^{ns}	0.14 ^{ns}	1.00***	1.00 ***	−1.00 ***	−0.80 **	−0.87 **
α-Phellandrene	0.99 ***	−0.94 ***	0.24^{ns}	0.20 ^{ns}	−0.28 ^{ns}	0.44 ^{ns}	−0.64 ^{ns}
α-Terpinene	−0.33 ^{ns}	0.17 ^{ns}	−0.86**	−0.85 **	0.87 **	0.50 ^{ns}	0.88 **
Limonene	0.99 ***	−1.00 ***	−0.07^{ns}	−0.12 ^{ns}	0.04 ^{ns}	0.70 *	−0.37 ^{ns}
1.8-Cineole	−0.48 ^{ns}	0.31 ^{ns}	−0.92***	−0.90 **	0.93 ***	0.45 ^{ns}	1.00 ***
(*Z*)-β-Ocimene	−0.78 *	0.88 **	0.55^{ns}	0.59 ^{ns}	−0.52 ^{ns}	−0.96 ***	−0.12 ^{ns}
(*E*)-β-Ocimene	−0.75 *	0.87 **	0.58^{ns}	0.62 ^{ns}	−0.55 ^{ns}	−0.95 ***	−0.16 ^{ns}
γ-Terpinene	−0.41 ^{ns}	0.24 ^{ns}	−0.94***	−0.93 ***	0.96 ***	0.51 ^{ns}	0.99 ***
Terpinolene	0.88 **	−0.96 ***	−0.37^{ns}	−0.41^{ns}	0.33 ^{ns}	0.88 **	−0.08 ^{ns}
Linalool	0.99 ***	−1.00 ***	−0.02^{ns}	−0.06^{ns}	−0.02 ^{ns}	0.65 ^{ns}	−0.43 ^{ns}
Camphor	0.82 **	0.82 ***	-0.50^{ns}	-0.54 ^{ns}	0.47 ^{ns}	0.94 ***	0.07 ^{ns}
Borneol	0.96 ***	0.96 ***	0.35^{ns}	0.40 ^{ns}	−0.32 ^{ns}	−0.87 **	0.10 ^{ns}
Terpinen-4-ol	0.71 *	−0.83 **	−0.64^{ns}	−0.67 *	0.61 ^{ns}	0.98 ***	0.23 ^{ns}
α-Terpineol	0.07 ^{ns}	−0.26 ^{ns}	−0.99***	−0.99 ***	0.98 ***	0.86 **	0.81 **
(−)-Bornyl acetate	0.88 **	−0.95 ***	−0.40^{ns}	−0.44 ^{ns}	0.36 ^{ns}	0.89 **	−0.05 ^{ns}
α-Cubebene	0.85 **	−0.74 *	0.60^{ns}	0.56 ^{ns}	−0.63 ^{ns}	0.06 ^{ns}	−0.89 **
α-Ylangene	−0.24 ^{ns}	0.42 ^{ns}	0.94***	0.96 ***	−0.93 ***	−0.94 ***	−0.70 *
α-Copaene	0.06 ^{ns}	−0.24 ^{ns}	−0.99***	−0.99 ***	0.98 ***	0.85 **	0.82 **
β-Cubebene	−0.15 ^{ns}	0.33 ^{ns}	0.97***	0.98 ***	−0.96 ***	−0.90 **	−0.77 *
β-Bourbonene	0.13 ^{ns}	−0.31 ^{ns}	−0.98***	−0.99 ***	0.97 ***	0.89 **	0.78 *
α-Bergamotene	−0.15 ^{ns}	0.33 ^{ns}	0.97***	0.98 ***	−0.96 ***	−0.89 **	−0.77 *
α-Gurjunene	0.75 *	−0.86 **	−0.60^{ns}	−0.63^{ns}	0.57 ^{ns}	0.97 ***	0.18 ^{ns}
trans-Caryophyllene	0.99 ***	−0.96 ***	0.20^{ns}	0.15 ^{ns}	−0.24 ^{ns}	0.48 ^{ns}	−0.61 ^{ns}
Aromadendrene	−0.25 ^{ns}	0.42 ^{ns}	0.94***	0.96 ***	−0.93 ***	−0.94 ***	−0.70 *
β-Farnesene	0.63 ^{ns}	−0.48 ^{ns}	0.83**	0.80 **	−0.85 **	−0.28 ^{ns}	−0.99 ***
α-Humulene	−0.96 ***	0.89 **	-0.37^{ns}	−0.32 ^{ns}	0.40 ^{ns}	−0.32 ^{ns}	0.74 *
allo-Aromadendrene	−.01 ^{ns}	0.20 ^{ns}	0.99***	1.00 ***	−0.99 ***	−0.83 **	−0.85 **
β-Selinene	0.03 ^{ns}	−0.47 ^{ns}	−0.92***	−0.94 ***	0.91 ***	0.96 ***	0.66 ^{ns}
Germacrene D	−0.06 ^{ns}	−0.25 ^{ns}	−0.99***	−0.99 ***	0.98 ***	0.86 **	0.82 **
γ-Muurolene	−0.37 ^{ns}	0.53 ^{ns}	0.89**	0.92 ***	−0.87 **	−0.97 ***	−0.60 ^{ns}
δ-Cadinene	−0.36 ^{ns}	0.53 ^{ns}	0.89**	0.91 ***	−0.88 **	−0.97 ***	−0.61 ^{ns}
Spathulenol	−0.39 ^{ns}	−0.22 ^{ns}	−0.95***	−0.93 ***	0.96 ***	0.53 ^{ns}	0.99 ***
Caryophyllene oxide	−0.57 ^{ns}	−0.71 *	−0.75*	−0.77 *	0.73 *	0.99 ***	0.39 ^{ns}
Viridiflorol	−0.94 ***	0.86 **	−0.43 ^{ns}	−0.39 ^{ns}	0.46 ^{ns}	−0.25 ^{ns}	0.78 *
Calarene	−0.11 ^{ns}	−0.07 ^{ns}	−1.00 ***	−1.00 ***	1.00 ***	0.75 ^{ns}	0.91 ***
Manoyl oxide	0.03 ^{ns}	−0.22 ^{ns}	−0.99 ***	−1.00 ***	0.99 ***	0.84 **	0.83 **

Manool	−0.02 ns	−0.20 ns	−0.99 ***	−1.00 ***	0.99 ***	0.83 **	0.84 **
Sclareol	−0.87 **	0.73 *	−0.58 ns	−0.54 ns	0.61 ns	−0.08 ns	0.88 **

ns Not significant. * Significant at $p < 0.05$. ** Significant at $p < 0.01$. *** Significant at $p < 0.001$.

Reference

[1] Ibraliu, A.; Doko, A.; Hajdari, A.; Gruda, N.; Šatović, Z.; Cvetkovikj Karanfilova, I.; Stefkov, G. Essential oils chemical variability of seven populations of *Salvia officinalis* L. in North of Albania. *Maced. J. Chem. Chem. Eng.* **2020**, *39*, 31.

[2] Schmiderer, C.; Torres, P.; Novak, J. Proof of geographical origin of Albanian sage by essential oil analysis. Biochem. *Syst. Ecol.* 2013, *51*, 70–77.

[3] Jug-Dujaković, M.; Ristić, M.; Pljevljakušić, D.; Dajić-Stevanović, Z.; Liber, Z.; Hančević, Z.; Radić, T.; Šatović,Z. High diversity of indigenous populations of Dalmatian sage (*Salvia officinalis* L.) in essential-oil Composition. *Chem. Biodiv*. **2012**, *9*, 2309–2323.

[4] Velickovic, D. T.; Ristic, M. S.; Randjelovic, N. V.; & Smelcerovic, A. A. Chemical composition and antimicrobial characteristic of the essential oils obtained from the flower, leaf and stem of *Salvia officinalis* L. originating from southeast Serbia. *J. Essent. Oil Res.* **2002**, *14*, 453–458.

[5] Couladis, M., Tzakou, O., Mimica-Dukić, N., Jančić, R., & Stojanović, D. Essential oil of *Salvia officinalis* L. from Serbia and Montenegro. *Flavour Fragr. J.* **2002**, *17*, 119–126.

[6] Lakušić, B.; Ristić, M.; Slavkovska, V.; Stojanović, D.; & Lakušić, D. Variations in essential oil yields and compositions of *Salvia officinalis* (Lamiaceae) at different developmental stages. *Botanica Serbica* **2013**, *37*, 127–139.

[7] Cvetkovikj, I.; Stefkov, G.; Karapandzova, M.; Kulevanova, S.; Šatović, Z. Essential oils and chemical diversity of Southeast European populations of *Salvia officinalis* L. *Chem. Biodiv*. **2015**, *12*, 1025–1039.

[8] Stešević, D.; Ristić, M.; Nikolić, V., Nedović, M.; Caković, D.; & Šatović, Z. Chemotype diversity of indigenous Dalmatian sage (*Salvia officinalis* L.) populations in Montenegro. *Chem. Bio.* **2014**, *11*, 101–114.

[9] Pace, L., & Piccaglia, R. Characterization of the essential oil of a wild Italian endemic sage: *Salvia officinalis* L. var. *angustifolia* Ten (Labiatae). *J. Essent. Oil Res.* **1995**, *7*, 443–446.
